# YKL-40 expression in chronic obstructive pulmonary disease: relation to acute exacerbations and airway remodeling

**DOI:** 10.1186/s12931-016-0338-3

**Published:** 2016-03-24

**Authors:** Tianwen Lai, Dong Wu, Min Chen, Chao Cao, Zhiliang Jing, Li Huang, Yingying Lv, Xuanna Zhao, Quanchao Lv, Yajun Wang, Dongming Li, Bin Wu, Huahao Shen

**Affiliations:** Department of Respiratory and Critical Care Medicine, Affiliated Hospital, Institute of Respiratory Diseases, Guangdong Medical College, Zhanjiang, China; Department of Respiratory and Critical Care Medicine, Second Affiliated Hospital, Institute of Respiratory Diseases, Zhejiang University School of Medicine, Hangzhou, China; Department of Respiratory Medicine, Ningbo First Hospital, Ningbo, China; Department of pathology, Affiliated Hospital, Guangdong Medical College, Zhanjiang, China; Department of pathology, Zhejiang University School of Medicine, Hangzhou, China

**Keywords:** Chronic obstructive pulmonary disease, CHI3L1, YKL-40, Exacerbation, Disease severity

## Abstract

**Background:**

Recent studies suggest that YKL-40, also called chitinase-3-like-1 protein, has been implicated in the pathogenesis of various inflammatory diseases. It is currently unknown, however, whether YKL-40 plays a role in acute exacerbations of chronic obstructive pulmonary disease (AECOPD) and airway remodeling.

**Methods:**

We evaluated serum YKL-40 levels in patients with AECOPD (*n* = 37) and stable COPD (*n* = 44), as well as in controls (*n* = 47). The association between YKL-40 expression and airway remodeling was analyzed. The effects of YKL-40 on collagen synthesis of primary human lung fibroblasts were also evaluated.

**Results:**

Serum YKL-40 levels were elevated at AECOPD onset as compared to stable disease (median [interquartile range], 78.6 [52.3–122.2] ng/ml versus 46.7 [31.2–75.5] ng/ml; *p* = 0.0005). The ideal cutoff point for distinguishing patients with AECOPD from those with stable COPD was 64.7 ng/ml (AUC: 0.71; 95%CI: 0.596 to 0.823). YKL-40 expression correlated with airflow obstruction, C-reactive protein, and collagen deposition. Stimulation with YKL-40 promoted collagen production in lung fibroblasts through ERK- and p38-dependent mechanisms.

**Conclusions:**

YKL-40 expression is up-regulated in patients with COPD and correlates with exacerbation attacks and may contribute to airway remodeling by acting on lung fibroblasts. The current data may provide insight into the underlying pathogenesis of COPD, in which YKL-40 has an important pathogenic role.

**Trial registration:**

ChiCTR-OCC-13003567

**Electronic supplementary material:**

The online version of this article (doi:10.1186/s12931-016-0338-3) contains supplementary material, which is available to authorized users.

## Background

Acute exacerbations of chronic obstructive pulmonary disease (AECOPD) are common events that often lead to hospital admissions, increased healthcare costs [1]. During exacerbation, COPD patients experience a worsening of symptoms that coincides with accelerated decline in lung function, resulting in a decrease in quality of life. Airway inflammation plays a pivotal role in the pathogenesis of AECOPD. However, methods used in clinical practice are not appropriate for the evaluation of airway inflammation [2, 3]. For example, spirometry is used to monitor disease activity, but it has been shown that spirometry is not closely associated with the levels of inflammation. Thus, identification of novel biomarkers associated with pathophysiologic changes in COPD is necessary to improve the clinical management of COPD for the benefit of the patients. Recently, several questions have been raised about the role of the chitinase-like protein YKL-40 in chronic bronchial inflammation. YKL-40 (also known as chitinase 3-like 1 (CHI3L1)) binds to the ubiquitously expressed chitin but lacks chitinase activity. Previous studies have demonstrated that YKL-40 is associated with various pathologic conditions that are characterized by aberrant cell growth, tissue inflammation and remodeling, such as asthma, idiopathic pulmonary fibrosis (IPF) and allergic rhinitis [4–15]. However, it is currently unknown whether YKL-40 plays a role in AECOPD.

Airway remodeling is another prominent pathophysiologic feature of COPD, which is characterized by thickening of the airway wall with increased collagen deposition [16]. The mechanisms underlying its development have not been fully elucidated. The extent of airway wall thickening is associated with disease progression, and this thickening is the major cause of decreased lung function in COPD as remodeling reduces airflow and distensibility [17–19]. Previous studies indicated that serum YKL-40 levels were increased in severe asthma patients and were correlated positively with the thickness of the subepithelial basement membrane [8, 20–23]. Furuhashi et al. demonstrated that increased expression of YKL-40 was involved in tissue remodeling and fibrosis in IPF patients [9]. Létuvé et al. suggested that YKL-40 may influence extracellular matrix deposit and turnover by inducing metal matrix proteinase (MMP)-9 production by alveolar macrophages [12]. These data suggest that YKL-40 contributes to tissue remodeling in various human diseases. However, there is no evidence that YKL-40 is involved in airway remodeling in COPD. Lung fibroblasts have been shown to contribute to airway remodeling in airway diseases through synthesis and secretion of the main components of the extracellular matrix (ECM), such as proteoglycans and collagens [17]. Park et al. showed that YKL-40 induced the increased production of transforming growth factor (TGF) beta1, MMP-9 and collagen production in human nasal mucosa fibroblast [24]. Recklies et al. also showed that YKL-40 was preferentially expressed in areas with active fibrogenesis in patients with hepatic fibrosis, where it may act synergistically with insulin-like growth factor I to stimulate the growth of fibroblasts [14, 25]. However, whether YKL-40 participates in the onset of deposition of ECM and fibrosis of the small airways in patients with COPD has not been explored.

In the present study, we hypothesized that the up-regulation of YKL-40 expression is more pronounced in more severe forms of COPD and could induce airway remodeling by acting on human lung fibroblasts. Firstly, we investigated the expression of YKL-40 in patients with COPD and identified its correlation to acute exacerbation, disease severity (e.g., lung function, arterial blood gases) and airway remodeling. In addition, we evaluated the proliferation, transformation and collagen production from primary human lung fibroblasts in vitro after stimulation with YKL-40. Finally, the potential mechanism of YKL-40 action on collagen production in human lung fibroblasts was explored.

## Methods

### Study population

From October 2013 to November 2014, a total of 81 patients with COPD as defined by the Global Initiative for Chronic Obstructive Lung Disease guidelines (GOLD) guidelines [1], who had a history of chronic respiratory symptoms, such as cough and sputum with or without breathlessness, had a postbronchodilator forced expiratory volume in 1 s (FEV_1_)/forced vital capacity (FVC) ratio of less than 0.7 were recruited. Exclusion criteria were as follows: any chronic cardiopulmonary disease other than COPD (including asthma); received oral or intravenous corticosteroids or any other anti-inflammatory drugs in the preceding four weeks, given the possibility that the anti-inflammatory drugs may be able to suppress the elevation of pulmonary YKL-40 levels to confound the results [26]; and an inability to give written informed consent or cooperate with the study investigators. We also recruited 47 age-matched healthy subjects with normal spirometry from the communities surrounding our hospital to serve as controls. They were free of respiratory tract infection in the four weeks prior to the study. The characteristic of the patients and controls are shown in Table 1. COPD patients (*n* = 81) were divided into a stable group (*n* = 44) and an exacerbation group (*n* = 37). The division was based upon the status of the patients at the time of the initial visit and those that were experiencing an exacerbation at that time point compared to those that were not. Stable COPD was defined as no change in their treatment course for four or more weeks and also had no evident acute exacerbations of COPD during that same time period [1]. AECOPD was defined as an event in the natural course of the disease characterized by a change in the patient’s baseline dyspnea, cough, and/or sputum that is beyond normal day-to-day variations, is acute in onset, and may warrant a change in regular medication in a patient with underlying COPD [1]. The patients with AECOPD were followed up, and post-exacerbation samples were collected when the patients were on their usual COPD treatment and at their baseline respiratory state.

**Table 1:** Baseline characteristic of subjects

		Patients with COPD			*P* value	
	Controls (*n* = 47)	All (*n* = 81)	Stable (*n* = 44)	Exacerbation (*n* = 37)	Control vs. all patients	Stable vs. exacerbation
Age, yrs	57.7 ± 1.5	58.3 ± 1.0	56.8 ± 1.3	60.0 ± 1.5	0.402	0.116
Male/female, n	28/19	54/27	27/17	27/10	0.297	0.270
Smoking history, packs/year	37.0 ± 1.7	42.9 ± 1.2	41.6 ± 1.1	44.3 ± 2.3	0.067	0.258
Smoking status Never/current/former, n	25/12/10	0/49/32	0/28/16	0/21/16	0.420	0.528
FEV_1_/FVC, %	82.9 ± 0.8	53.1 ± 1.4	56.1 ± 1.8	49.7 ± 2.2	<0.001	0.027
FEV_1_, % predicted	96.6 ± 1.6	54.7 ± 2.4	61.4 ± 3.2	46.7 ± 3.3	<0.001	0.002
PaO_2_, mmHg	N/D	74.2 ± 1.0	77.5 ± 1.0	70.3 ± 1.5	―	<0.001
PaCO_2_, mmHg	N/D	43.1 ± 0.6	40.6 ± 0.7	45.9 ± 0.9	―	<0.001
CRP, mg/L	3.5 ± 0.3	36.7 ± 3.6	14.2 ± 1.1	63.5 ± 4.5	<0.001	<0.001
Severity of COPD^a^	N/A				―	0.489
GOLD I/II, n (%)		47 (58.0)	24 (54.5)	23 (62.2)		
GOLD III/IV, n (%)		34 (42.0)	20 (45.5)	14 (37.8)		
COPD treatments, n (%)	N/A				―	0.935
ICS/LABA		58 (71.6)	32 (72.7)	26 (70.3)		
SABA or SAMA		71 (87.7)	38 (86.4)	33 (89.2)		
Theophylline		51 (63.0)	29 (65.9)	22 (59.5)		

Data are presented as mean ± SEM, unless otherwise stated

^a^COPD severity was graded into GOLD I, Mild (FEV_1_ ≥ 80 % predicted); GOLD II, Moderate (50 % ≤ FEV_1_ < 80 % predicted); GOLD III, Severe (30 % ≤ FEV_1_ < 50 % predicted); and GOLD IV, Very severe (FEV_1_ < 30 % predicted) following the GOLD

*FEV*_*1*_ forced expiratory volume in 1 s, *FVC* forced vital capacity, *PaO*_*2*_ arterial partial oxygen pressure, *PaCO*_*2*_ arterial partial carbon dioxide pressure, *CRP* C reactive protein, *COPD* chronic obstructive pulmonary disease, *GOLD* Global Initiative for Chronic Obstructive Lung Disease, *ICS* inhaled corticosteroid, *LABA* long-acting β agonist, *SABA* short-acting β agonist, *SAMA* short-acting muscarinic agonist, *N/D* not done, *N/A* not applicable

The study protocol was approved by the Ethics of Research Committee of the Medical College of Guangdong and was registered on the Chinese Clinical Trial Database (ChiCTR-OCC-13003567). Written informed consent was obtained from all participants.

### Sample collection

To investigated the expression of YKL-40 in the lung tissue and identified its correlation to airway remodeling, lung tissue specimens were obtained from patients who were undergoing lung lobectomy for localized lung carcinoma (Additional file 1: Table S1). Specimens were dissected at a distance of ≥ 5 cm away from the tumor.

### Laboratory measurements

Pulmonary function tests were performed according to American Thoracic Society (ATS) guidelines either on the same day as the bronchoscopy or on the day that the serum samples were collected [27]. Blood gas analysis was performed using a gas analyzer (IL GEM Premier 3000, USA). CRP was performed using ARRAY 360 automatic protein analyzer (BECKMAN, USA). The reference value of serum CRP concentration was 0–10 mg/L. YKL-40 levels were measured using commercially available enzyme-linked immunosorbent assay (ELISA) kits (Uscn Life Science Inc., Wuhan, China). The minimum detection limit of the YKL-40 assay was 13.1 pg/ml.

### Immunohistochemistry

Details on the methods used to make these measurements are provided in the Additional file 1. Quantitative measurements of YKL-40 positive cells in the lung tissue were performed according to previously described methods [28]. YKL-40 positive cells were expressed as a percentage of total cells. The intraobserver error was assessed by performing three independent counts on the same section on separate occasions. Sections were examined using a light microscope (BX51; Olympus, Japan) and quantified by the Image Pro 6.1 software (Media Cybernetics).

### Quantitation of peribronchial collagen deposition

Peribronchial collagen deposition was detected by Masson trichrome staining. The area of peribronchial Masson trichrome staining (blue color) was visualized and is quantified by the software as a percentage of the total band area as previously described [29].

### Human fibroblasts isolation and stimulation

Primary human lung fibroblasts were isolated from lung tissue obtained from donors undergoing resection for localized lung carcinoma who gave informed consent, as described previously [17]. The available clinical characteristics of donors, including age, pack-years, and lung function, are provided in Additional file 1: Table S1. All experiments were carried out using cells between passage 3 and 6. Details on isolation and cultivation of human lung fibroblasts are also provided in the Additional file 1.

### Cell viability assays, migration and proliferation

To investigate cell viability, cells were seeded into a 96-well plate at a density of 1 × 10^4^ cells/well. The cells were treated with different doses of recombinant human YKL-40 protein (R&D Systems, Minneapolis, USA) for 48 h. A CCK-8 assay (Liankebio, Hangzhou, China) was used to determine cell viability according to the manufacturer’s instructions. A ‘scratch-wound’ assay was used to assess fibroblast migration fibroblast as described previously [30]. Full details of this method are available in the Additional file 1.

### Western blotting assay

Western blot analysis was used to detect changes in collagen type I, collagen type III, α-SMA, ERK, phosphorylated ERK (p-ERK), p38 and phosphorylated (p-p38) (Cell Signaling Technology, USA) as previously described [31].

### Statistical analysis

Data were presented as the mean ± SEM, unless otherwise stated. Statistical analysis was performed using SPSS 17.0 (SPSS, Chicago, IL, USA) and GraphPad Prism 5.0 software (GraphPad Software Inc., San Diego, CA, USA). Statistical significance was set at a *p* value < 0.05. Full details are available in the Additional file 1.

## Results

### Clinical data

Table 1 shows the main clinical and functional characteristics of the subjects in the study. There was no significant difference in age, gender or smoking status. Compared with patients in the stable group and control group, those in the exacerbation group had lower lung function (*p* = 0.002) and PaO_2_ levels (*p* < 0.001) but higher PaCO_2_ levels (*p* < 0.001) and serum CRP levels (*p* < 0.001). Similar proportions of subjects were receiving medications in both groups (*p* = 0.935).

### Serum YKL-40 levels were elevated during an AECOPD

**Fig. 1:**
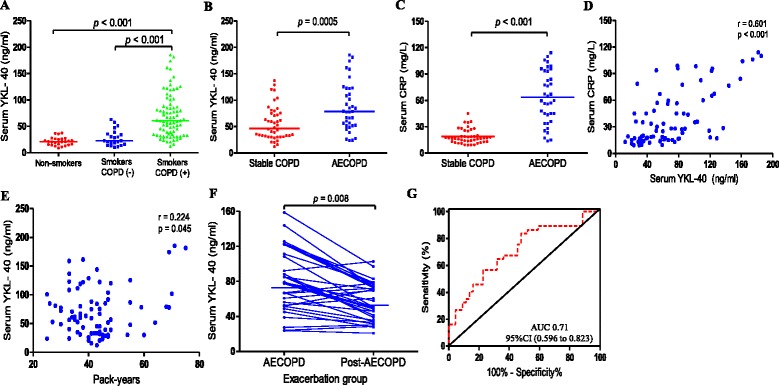
Serum YKL-40 levels in patients with COPD and controls. Serum YKL-40 was increased in smokers with COPD (defined as COPD (+)) compared with smokers without COPD (defined as COPD (−)) and non-smokers (**a**); When patients were stratified according to exacerbation attacks, the serum YKL-40 and C-reactive protein (CRP) levels in patients in the exacerbation group were higher than those in the stable group (**b, c**); Spearman rank correlation coefficient showed that YKL-40 was positively associated with CRP (**d**); Borderline significance was evident between YKL-40 concentrations and pack-years (*r* = 0.224, *p* = 0.045) (**e**); Patients in the exacerbation group, after acute exacerbations, demonstrated decreased serum YKL-40 levels compared with those during AECOPD (**f**); Receiver operating characteristic (ROC) curve for distinguishing patients with AECOPD from those with stable COPD. The area under the ROC curve was 0.71 (95%CI: 0.596 to 0.823). Horizontal bars represent median values (**g**)

The serum YKL-40 levels for patients in smokers with COPD were higher than those in smokers without COPD (median [interquartile range], 60.80 [34.7–90.1] ng/ml versus 22.7 [15.6–36.4] ng/ml; *p* < 0.001) and those in never-smoker individuals (78.60 [52.3–90.11] ng/ml versus 20.9 [15.2–26.3] ng/ml; *p* < 0.001) (Fig. 1a). The serum YKL-40 and CRP levels of COPD patients in the exacerbation group were higher than those in stable group (YKL-40: 78.6 [52.3–122.2] ng/ml versus 46.7 [31.2–75.5] ng/ml; *p* = 0.0005; CRP: 60.0 [39.9–93.4] mg/L versus 16.9 [13.0–23.8] mg/L; *p* < 0.001) (Fig. 1b, c). Moreover, serum levels of YKL-40 were positively correlated with CRP (*r* = 0.601, *p* < 0.001) (Fig. 1d). Spearman rank correlation coefficient showed that borderline significance was evident between YKL-40 concentrations and pack-years (*r* = 0.224, *p* = 0.045) (Fig. 1e). During the follow-up visit, five AECOPD patients were lost to follow-up because they refused to continue participating. Finally, 32 subjects completed the study and were included in the data analyses. We found that patients in the exacerbation group, after acute exacerbations, demonstrated decreased serum YKL-40 levels compared with those during AECOPD (54.3 [36.4–74.4] ng/ml versus 77.7 [50.9–113.9] ng/ml; *p* = 0.008) (Fig. 1f). The ideal cutoff point for distinguishing patients with AECOPD from those with stable COPD was 64.7 ng/ml (sensitivity, 64.8 %; specificity, 71.2 %; AUC, 0.71; 95%CI: 0.596 to 0.823) (Fig.1g). YKL-40 levels may be affected by age and gender. Thus, we further analyzed the between-group differences following adjustment for age and gender using multiple regression analyses. We found that the AECOPD patients had significantly higher serum YKL-40 levels than stable COPD patients, even after adjustment for sex, age. In addition, women had lower serum YKL-40 levels, whereas older age was associated with higher serum YKL-40 concentrations (Additional file 1: Table S2).

### Serum YKL-40 levels in COPD patients were correlated with clinical parameters

Spearman’s rank correlation analysis showed that serum YKL-40 levels in COPD patients were correlated negatively with FEV_1_ and PaO_2_. However, there were no significant correlations between serum YKL-40 levels and other clinical parameters, such as the FEV_1_/FVC or PaCO_2_ (Table 2).

**Table 2:** Correlation between serum YKL-40 levels and clinical parameters^a^

Parameters	Serum YKL-40					
	Stable (*n* = 44)		Exacerbation (*n* = 37)		All (*n* = 81)	
	*r*_*s*_	*P* value	*r*_*s*_	*P* value	*r*_*s*_	*P* value
FEV_1_, % of predicted	−0.399	0.007	−0.440	0.006	−0.442	<0.001
FEV_1_/FVC, %	−0.156	0.356	−0.01	0.944	−0.201	0.072
CRP, mg/L	0.507	<0.001	0.624	<0.001	0.601	<0.001
PaO_2_, mmHg	−0.349	0.020	−0.60	<0.001	−0.556	<0.001
PaCO_2_, mmHg	0.018	0.917	0.087	0.573	0.185	0.098

^a^Spearman’s rank order method. *FEV*_*1*_ forced expiratory volume in 1 s, *FVC* forced vital capacity, *CRP* C reactive protein, *PaO*_*2*_ arterial partial oxygen pressure, *PaCO*_*2*_ arterial partial carbon dioxide pressure

### Increased YKL-40 expression was positively correlated with collagen deposition

**Fig. 2:**
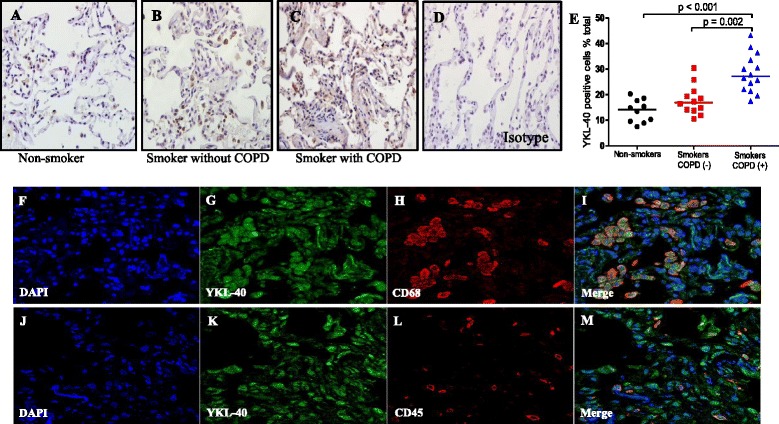
YKL-40 was expression in lung tissues and localized within macrophages and neutrophils. Very faint staining for YKL-40 was observed in the non-smokers (**a**); In the smokers without COPD, there were more YKL-40 positive cells in the lung parenchyma (**b**); Smokers with COPD had considerably more YKL-40 positive cells staining in the lung parenchyma (expressed as a percentage of total cells) (**c**); No immunostaining was observed in control isotype IgG-treated tissue sections (**d**); Quantification of YKL-40 expression in lung tissues (**e**); Confocal microscopy showed localization of YKL-40 (green fluorescence) within CD68 or CD45 positive cells (red fluorescence) of the tissue sections (**f-i** and **j-m**, respectively). (all images are × 400 magnification). Horizontal bars represent median values

**Fig. 3:**
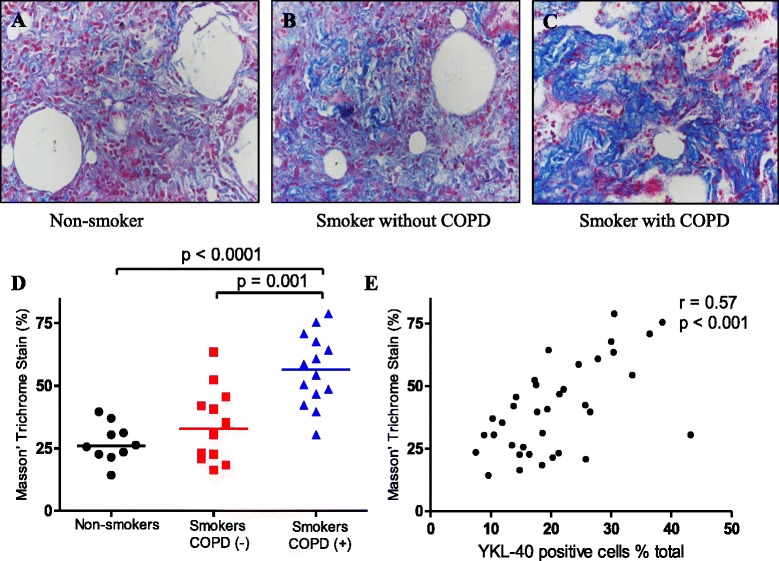
Collagen deposition in small airways from non-smokers, smokers without COPD and smokers with COPD. Photomicrographs of blue staining showing collagen expression around the small airway walls from non-smokers (**a**), smokers without COPD (**b**), and smokers with COPD (**c**) (all images are × 400 magnification); Measurement of collagen deposition in small airways. Smokers with COPD showed increased collagen deposition compared to both the non-smokers, smokers without COPD (**d**); Collagen deposition was positively correlated with serum YKL-40 levels (*r* = 0.57; *p* < 0.001) (**e**). Horizontal bars represent median values

Examination of lung tissue sections from patients who were undergoing lung lobectomy for peripheral carcinoma showed that smokers with COPD, the percentage of YKL-40 positive cells (27.1 [21.9–34.2]%) was significantly increased than those without COPD (16.8 [13.9–20.8]%; *p* = 0.002) and non-smokers (14.2 [9.4–17.9]%; *p* < 0.001) (Fig. 2a–c, e). No immunostaining was observed in control isotype IgG-treated tissue sections (D). Detailed examination of the cellular sources of YKL-40 in lung tissue revealed that a high level of expression of YKL-40 in macrophages and neutrophils (Fig. 2f–m). Collagen deposition in the lungs was increased in smokers with COPD (56.5 [45.6–68.5]%) compared to smokers without COPD (33.2 [21.2–44.9]%; *p* = 0.001) and non-smokers (25.9 [22.3–32.7]%; *p* < 0.001) (Fig. 3a-d). Furthermore, collagen deposition correlated with YKL-40 expression in lung tissues (*r* = 0.57; *p* < 0.001) (Fig. 3e).

### YKL-40 can stimulate the proliferation, differentiation and collagen synthesis in human lung fibroblast

**Fig. 4:**
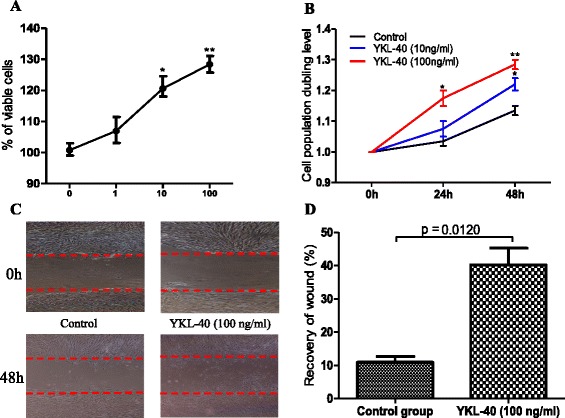
The effect of YKL-40 treatment on the proliferation and migration of human lung fibroblast cells. The cells were treated with 0, 10 or 100 ng/ml YKL-40 for 48 h, then resuspended and counted (**a**); The cells were treated with 0, 10 or 100 ng/ml YKL-40 for 0 h, 24 h and 48 h, then cell viability was measured with CCK-8 assay. Increased number of YKL-40-treated fibroblasts versus control fibroblasts was observed (**b**). Representative images of human lung fibroblast cells treated with YKL-40 or untreated, at time 0 and after 48 h of incubation were shown. Increased fibroblast migration was observed in YKL-40-treated fibroblasts versus control fibroblasts (**c**). Results are expressed as percentage of recovered wound area (**d**). Results were expressed as mean ± SEM (*n* = 4 per group) of three independent experiments. *p < 0.05, **p < 0.01 compared with basal

**Fig. 5:**
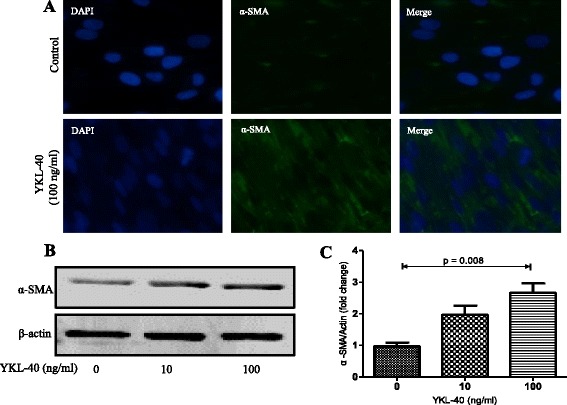
The effect of YKL-40 treatment on α-SMA protein expression in lung fibroblast cells. Lung fibroblast cells were treated with 0, 10 or 100 ng/ml YKL-40 for 48 h. Measurements of α-SMA expression upon stimulation by YKL-40 as determined by immunofluorescence staining (**a**), and Western blot analysis (**b**); Analysis by densitometry of immunodetection of α-SMA (**c**). Results were expressed as mean ± SEM of three independent experiments

**Fig. 6:**
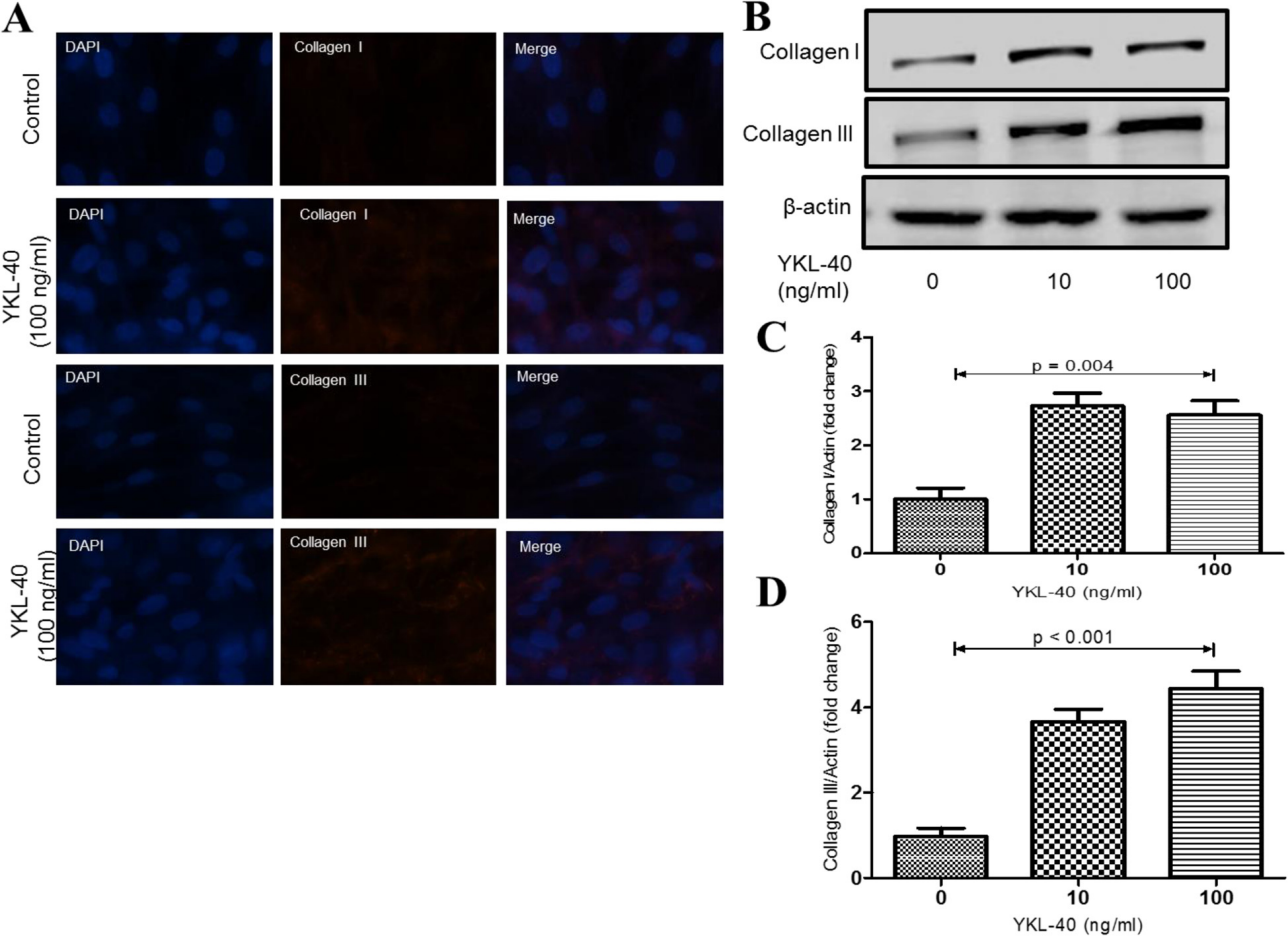
The effect of YKL-40 on collagen synthesis in lung fibroblast cells. Lung fibroblast cells were treated with 0, 10 or 100 ng/ml YKL-40 for 48 h. Collagen I and collagen III production were determined by immunofluorescence staining (**a**), and Western blot analysis (**b**); Analysis by densitometry of immunodetection of collagen I and collagen III (**c, d**). Results were expressed as mean ± SEM of three independent experiments

The migration of human lung fibroblast cells was evaluated by a scratch wound assay. Treatment with YKL-40 significantly increased the number of human lung fibroblast cells as compared to the untreated control (Fig. 4a-b). Treatment with YKL-40 also increased the migration capacity of human lung fibroblast cells compared with the untreated control (Fig. 4c-d). The upregulation of α-SMA expression is a characteristic marker for differentiation of lung fibroblasts to myofibroblasts [23]. Using immunofluorescence and western blotting, we detected the expression of α-SMA in human lung fibroblast cells after 48 h of treatment with YKL-40 (10 and 100 ng/ml) and found that it significantly increased α-SMA protein expression in a concentration-dependent manner compared with the untreated control cells (Fig. 5). Treatment with YKL-40 (100 ng/ml) also significantly increased the expression of collagen I and III production compared with untreated control fibroblast cells (Fig. 6).

### Increased phosphorylation of p-38 and ERK in YKL-40-induced collagen production

**Fig. 7:**
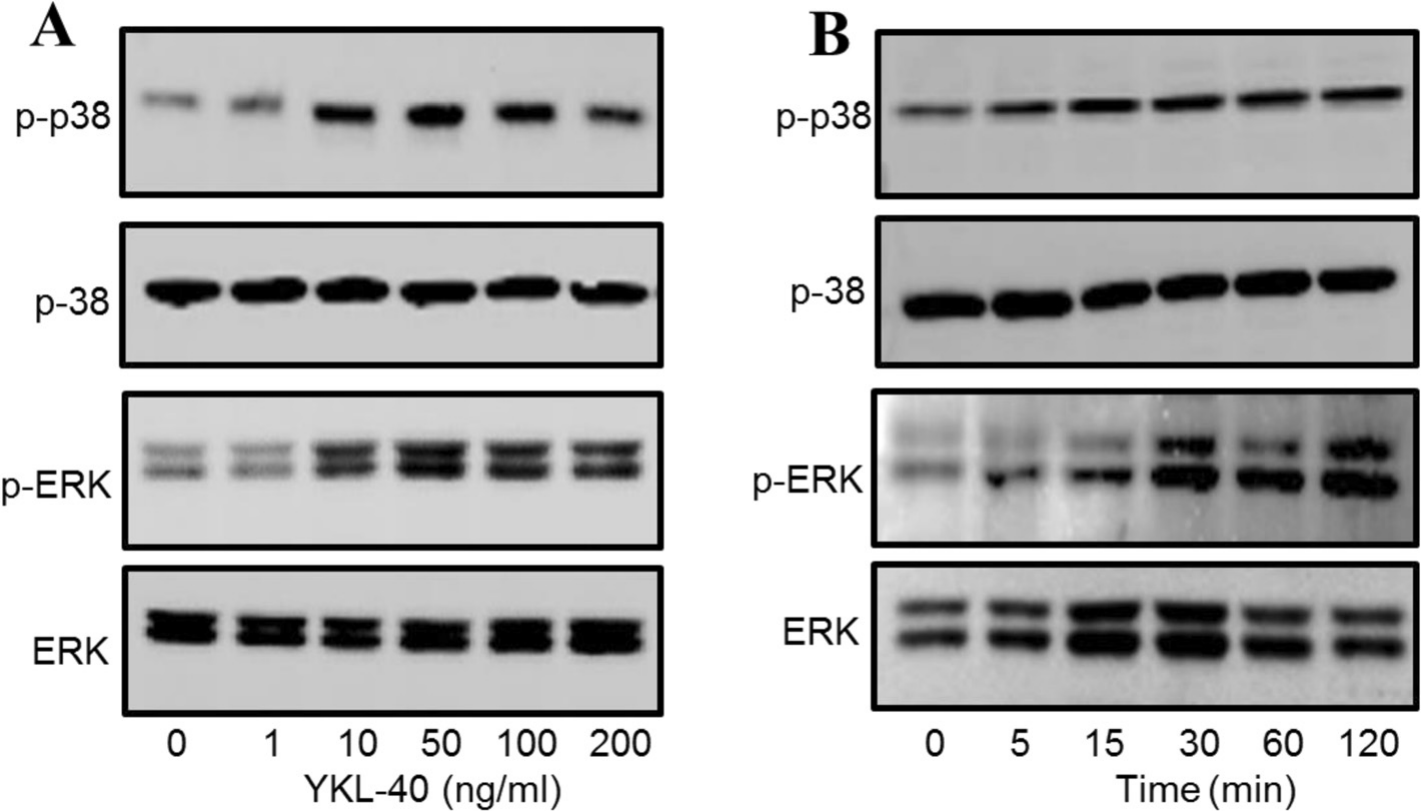
YKL-40 induced phosphorylation of extracellular signal related kinase (ERK), and p38 in lung fibroblast cells. Recombinant human YKL-40 protein activated ERK and p38 phosphorylaton in a dose-dependent manner (0, 1, 10, 50, 100 and 200 ng/ml for 2 h) (**a**); Stimulation of lung fibroblast cells with 50 ng/ml of recombinant human YKL-40 protein also induced phosphorylation of ERK and p38 in a time-dependent manner (0, 5, 15, 30, 60, 120 min) (**b**)

The mitogen-activated protein kinase (MAPK) family is well known to play a key role in mediating inflammatory responses [32]. To assess whether MAPK pathways are involved in YKL-40-induced collagen production, the effect of YKL-40 on activation of ERK and p38 was evaluated by western blotting. As shown in Fig. 7, stimulation of human lung fibroblast cells with YKL-40 resulted in a transient phosphorylation of ERK and p38. In addition, YKL-40 activated ERK and p38 phosphorylaton in a dose-dependent manner.

## Discussion

In the present study, we further explored the potential role of YKL-40 measurement in the management of COPD. Our study indicated that YKL-40 levels were increased in patients with COPD during exacerbation, and the elevated YKL-40 was associated positively with CRP and negatively with FEV_1_ and PaO_2_. Importantly, our findings revealed that the expression of YKL-40 in lung tissues of COPD patients was correlated with deposition of collagen in the airway walls and induced lung fibroblast activation, suggesting that a potential mechanism of small airway remodeling in COPD.

COPD is accompanied by systemic inflammation that occurs as a result of many mechanisms, particularly airway inflammation and smoking [33–35]. Previous studies indicated that YKL-40 was increased in many inflammatory diseases that were accompanied by tissue destruction. TNF-α stimulated YKL-40 synthesis in alveolar macrophages, and exposure of these cells to YKL-40 promoted the release of IL-8, monocyte chemoattractant protein (MCP)-1, macrophage inflammatory protein (MIP)-1α, and metalloproteinase-9 [12]. We found that serum YKL-40 levels were increased in COPD compared with smokers without COPD and non-smokers. When the COPD subjects were stratified, serum YKL-40 levels in the exacerbation group were higher than those in the stable group, which suggested that serum levels of YKL-40 correlated with COPD exacerbation attacks. Previous study by Nordenbaek et al. has shown that serum YKL-40 reflects a different aspect of the inflammatory pulmonary process than conventional acute-phase proteins [36]. CRP is increased during bacterial infections which could be the cause of the exacerbations in the patients with COPD. CRP levels are useful in evaluating COPD exacerbation [37]. Consistent with previous studies, we found that the serum YKL-40 levels and CRP were elevated in patients with AECOPD. Moreover, the serum YKL-40 levels correlated positively with serum CRP levels. These findings indicated that YKL-40 has a potential effect on the pathogenesis of inflammation of COPD and may serve as a specific serologic marker of granulocyte function at the site of tissue inflammation as a supplement to conventional acute-phase proteins [36]. Gumus et al. showed that high serum YKL-40 level is related to hypoxemia and hypoxia-related mediators may cause systemic inflammation in COPD [38]. We also found that serum YKL-40 levels correlated inversely with PaO_2_ as well as FEV_1_. However, borderline significance was evident between YKL-40 concentrations and pack-years. These findings are consistent with those reported by Matsuura and colleagues [10]. These results suggested that concentrations of circulatory YKL-40 in patients with COPD are profoundly affected by other local or systemic factors in addition to cigarette smoke (CS) exposure [10].

Peribronchiolar fibrosis is an important feature of COPD and is resulted from the increased extracellular matrix deposition [12]. Collagens are the classical components of the extracellular matrix. Collagens are synthetized primarily by fibroblasts as precursor molecules with the propeptides being cleaved during the process of secretion of the newly formed collagens [39]. YKL-40 has been shown to be a growth factor for mesenchymal cells that contributes to degradation of extracellular matrix and tissue remodeling [20–24]. Moreover, YKL-40 acts a chemoattractant for endothelial cells, and modulates vascular endothelial cell morphology by promoting the formation of branching tubules [9]. Previous studies indicated that YKL-40 promoted reticular basement membrane (RBM) thickening in severe asthma and contributed to tissue remodeling and fibrosis in IPF patients [9, 20–22]. Collectively, these data suggest that increased levels of YKL-40 contribute to the pathologic process of human diseases with tissue remodeling. Therefore, we further hypothesized that YKL-40 might promote collagen desposition and airway remodeling in COPD. We examined the expression of YKL-40 in small airways from smokers with COPD and controls by immunohistochemistry. There was a significant increase in YKL-40 expression in small airways of smokers with COPD and correlated closely with deposition of collagen. These data suggest that YKL-40 may be associated with small airway remodeling, a finding that could help elucidate the mechanism of small airway remodeling in COPD.

Recent studies have shown that YKL-40 is preferentially expressed in areas with active fibrogenesis in patients with hepatic fibrosis, where it may act synergistically with insulin-like growth factor I to stimulate the growth of fibroblasts [12]. YKL-40 may also contribute to fibrosis by modulating the rate of type I collagen fibril formation [40]. However, whether YKL-40 participates in the onset of fibrosis of the small airways in patients with COPD remains to be determined. On the basis of the above findings, we were prompted to further explore the role of YKL-40 in collagen production in human lung fibroblasts in vitro. As expected, we found that treatment with YKL-40 increased proliferation, migration, collagen secretion and α-SMA expression in human lung fibroblasts. Our results were consistent with previous work demonstrating that YKL-40 are able to increase ECM, such as proteoglycans and collagens in nasal mucosa fibroblast [24]. Given the role of YKL-40 in collagen production in human lung fibroblasts, we sought to determine the key signaling mechanisms by which this occurs. We demonstrated that MAPK signaling was required for YKL-40-induced collagen production in human lung fibroblasts. Taken together, we presume that YKL-40 could activate lung fibroblasts and its downstream MAPK pathway, and promote proliferation and collagen production, which may enhance the progression of small airway remodeling in COPD.

There are some limitations to this study that need to be considered. First, exposure to YKL-40 significantly up-regulated proliferation from fibroblasts obtained from non-smokers, smokers without COPD and smokers with COPD. Although there was a trend to up-regulated proliferation from fibroblasts obtained from smokers with COPD, there were no significantly difference among these different patient groups (Additional file 1: Figure S1). Due to the relatively small sample size, we could not precisely analyze the fibroblasts from these different patient groups behaved differently regarding their responses towards YKL-40. Future studies with larger sample sizes as well as animal experiments should be performed to clarify this issue as it may suggest a target for therapeutic intervention in future. Second, it is well known that tumors attract macrophages which are critical in influencing tumor growth and metastasis [41, 42]. Previous study showed that YKL-40 is synthesized by activated macrophages [43]. Although resected normal tissues more than 5 cm away from the tumor in our study, it should be noted that tumors attract macrophages which are critical in influencing tumor growth and metastasis and YKL-40 is synthesized by activated macrophages. Therefore, the expression of YKL-40 in lung tissues should be interpreted cautiously due to this limitation.

## Conclusions

In summary, the current findings demonstrate that elevated YKL-40 levels are associated with acute exacerbations and airway remodeling in patients with COPD. Moreover, the in vitro data show that YKL-40 may promote airway remodeling in COPD by acting on human lung fibroblasts. Overall, the current data may provide insight into the underlying pathogenesis of COPD, in which YKL-40 has an important pathogenic role.

## Additional file

Additional file 1: YKL-40 expression in chronic obstructive pulmonary disease: relation to acute exacerbations and airway remodeling. **Table S1.**
Characteristics of patients undergoing lung resection. **Table S2.** A multivariable linear regression model predicting the YKL-40 levels in patients with AECOPD and patients with stable COPD adjusted for sex, age. **Figure S1.** The effect of YKL-40 treatment on the proliferation of human lung fibroblast cells from non-smokers, smokers without COPD and smokers with COPD. (DOCX 60.7 kb)

## Abbreviations

COPD: chronic obstructive pulmonary disease
FEV_1_: forced expiratory volume in one second
FVC: forced vital capacity
GOLD: Chronic Obstructive Lung Disease guidelines
