# Supplementary material for: YKL-40 expression in chronic obstructive pulmonary disease: relation to acute exacerbations and airway remodeling
Source: Respir Res. 2016 Mar 24;17:31. doi: 10.1186/s12931-016-0338-3 (PMC4806429; doi:10.1186/s12931-016-0338-3)
Supplement: Additional file 1: — YKL-40 expression in chronic obstructive pulmonary disease: relation to acute exacerbations and airway remodeling. Table S1. Characteristics of patients undergoing lung resection. Table S2. A multivariable linear regression model predicting the YKL-40 levels in patients with AECOPD and patients with stable COPD adjusted for sex, age. Figure S1. The effect of YKL-40 treatment on the proliferation of human lung fibroblast cells from non-smokers, smokers without COPD and smokers with COPD. (DOCX 60.7 kb) [file 12931_2016_338_MOESM1_ESM.docx]

**Additional file 1**

**Title page**

**YKL-40 expression in chronic obstructive pulmonary disease: relation to acute exacerbations and airway remodeling**

Tianwen Lai^1,2†^, Dong Wu^1†^, Min Chen^1†^, Chao Cao^3†^, Zhiliang Jing^4^, Li Huang^5^, Yingying Lv^1^, Xuanna Zhao^1^, Quanchao Lv^1^, Yajun Wang^1^, Dongming Li^1^, Bin Wu^1*^ and Huahao Shen^2*^

^†^Equal contributors

^*^ Correspondence to Huahao Shen or Bin Wu. Email: huahaoshen@163.com (Huahao Shen); wubin621011@126.com (Bin Wu).

1. Department of Respiratory and Critical Care Medicine, Affiliated Hospital, Institute of Respiratory Diseases, Guangdong Medical College, Zhanjiang, China.

2. Department of Respiratory and Critical Care Medicine, Second Affiliated Hospital, Institute of Respiratory Diseases, Zhejiang University School of Medicine, Hangzhou, China.

3. Department of Respiratory Medicine, Ningbo First Hospital, Ningbo, China.

4. Department of pathology, Affiliated Hospital, Guangdong Medical College, Zhanjiang, China.

5. Department of pathology, Zhejiang University School of Medicine, Hangzhou, China.

***Immunohistochemistry***

The slides were incubated with either a rabbit polyclonal Ab against human YKL-40 (Uscn Life Science Inc., Wuhan, China) or a rabbit IgG (Santa Cruz Biotechnoloy) at the same concentration as the control isotype. Immunofluorescent staining was performed on paraffin-embedded lung tissue sections. The antibodies that were used in appropriate combinations were rabbit anti-human YKL-40 antibodies (Uscn Life Science Inc., Wuhan, China); rabbit anti-human Collagen I antibodies (Proteinthech, Chicago, USA); rabbit anti-human Collagen III antibodies (Proteinthech, Chicago, USA); mouse anti-human α-SMA antibodies (Abcam, Cambridge, UK); mouse anti-human CD68 antibodies (Abcam, Cambridge, UK); and mouse anti-human CD45 antibodies (Boster, Wuhan, China).

***Isolation of human lung fibroblasts*** [1]

Cells were obtained from proximal lung tissue containing small airways (< 1 mm) which were deemed to be free of tumour following pathological examination. This tissue was minced in 1-2 mm pieces into sterile Hanks Buffered Saline Solution (Hanks) and centrifuged for 5 minutes at 1000 rpm. Supernatant was aspirated and the tissue pellet was resuspended and plated onto tissue culture grade plastic flasks in 10% (vol/vol) FBS/2% antibiotics/DMEM. This was washed briefly in 70% ethanol followed by 3 changes of sterile Hanks solution. The epithelial layer was manually removed, followed by blunt dissection of airway smooth muscle bundles out of the section. Identification of fibroblasts was based on the expression of vimentin. Human lung fibroblasts were sub-cultured in six-well plates in 10% (vol/vol) FBS/2% antibiotics/DMEM at a density of 2.5×10^5^ cells/well. Cells were stimulated with/without YKL-40 (R&D Systems, USA) in a time (0h, 24h, 48h)- or concentration (1 ng/ml, 10 ng/ml, 100 ng/ml)-dependent manner.

***Cell viability assays, migration and proliferation*** [2]

Briefly, 2×10^5^ fibroblasts/well were seeded into 6-well plates and grown to confluence (37°C, 5%CO_2_). A pipette tip was used to scratch the monolayer to create a cell-free wound area. Untreated cells or cells treated with YKL-40 were allowed to migrate into this cell-free wound for 48h. The recovery of the wound area was quantified and expressed as the ratio of the recovered wound area to the initial wound area. Lung fibroblasts (5×10^4^ cells/well) were seeded into 12-well plates in DMEM medium (Hiclone, Utah, USA) with YKL-40 or without YKL-40 on day 0. On days 1 and 2, the cells were trypsinized and stained with trypan blue, and the number of viable cells was counted. All the experiments were repeated for three times.

***Statistical analysis***

YKL-40 levels, expressed as a median and an interquartile range, were not normally distributed. The nonparametric tests (Mann–Whitney U or Kruskal–Wallis) were used for comparisons between patient groups. Differences of parametric variables among different groups were assessed by one-way analysis of variance (ANOVA). The Spearman’s test was used for correlation analysis between the two data. To determine whether YKL-40 differentiated between stable COPD and AECOPD onset, the area under the curve (AUC) receiver operating characteristics (ROC) for YKL-40 was analyzed. Multivariate relationships were examined with linear regression models. For the model comparing levels between AECOPD patients and stable COPD patients, multiple regression analyses were performed after proper adjustments for age, gender.

**Refrences**

[1] Krimmer DI, Burgess JK, Wooi TK, Black JL, Oliver BG. Matrix proteins from smoke-exposed fibroblasts are pro-proliferative. *Am J Respir Cell Mol Biol* 2012;46:34-9.

[2] Liang CC, Park AY, Guan JL. In vitro scratch assay: a convenient and inexpensive method for analysis of cell migration in vitro. *Nat Protocols* 2007; 2: 329–333.

| **Table S1. Characteristics of patients undergoing lung resection** | | | | |
| --- | --- | --- | --- | --- |
|  | Non-smokers | Smokers without COPD | Smokers with COPD | *p* Value |
| Subjects, n | 10 | 12 | 14 |  |
| Age, yrs | 55.3 ± 2.5 | 56.8 ± 2.3 | 55.7 ± 1.9 | NS |
| Male/female, n | 6/4 | 9/3 | 10/4 | NS |
| Smoking history,  packs/year | 0 | 48.5 ± 2.1 | 58.3 ±4.1 | NS |
| FEV_1_/FVC, % | 82.6 ± 0.9 | 77.1 ± 1.2 | 59.8 ± 1.4 | < 0.001 |
| FEV_1_, % predicted | 100.4 ± 1.2 | 96.9 ± 1.5 | 67.1 ± 2.1 | < 0.001 |
| Data are presented as mean ± SEM, unless otherwise stated.  FEV_1_, forced expiratory volume in 1 second; FVC, forced vital capacity; COPD, chronic obstructive pulmonary disease; NS, non significant. | | | | |

| **Table S2. A multivariable linear regression model predicting the YKL-40 levels in patients with AECOPD and patients with stable COPD adjusted for sex, age** | | | |
| --- | --- | --- | --- |
| Variable | Regression Coefficient (*R*^2^ = 0.16) | 95%CI | P Value |
| Study category |  |  | 0.001 |
| Stable COPD group | 0 | – |  |
| AECOPD group | 0.38 | 14.0 to 49.3 |  |
| Age | 0.02 | – 0.88 to 1.091 | 0.828 |
| Sex |  |  | 0.299 |
| Male | 0 | – |  |
| Female | – 0.11 | – 30.6 to 9.5 |  |
| Constant | – | – 4.8 to 109.7 | 0.072 |
| AECOPD: Acute exacerbation of chronic obstructive pulmonary disease. | | | |


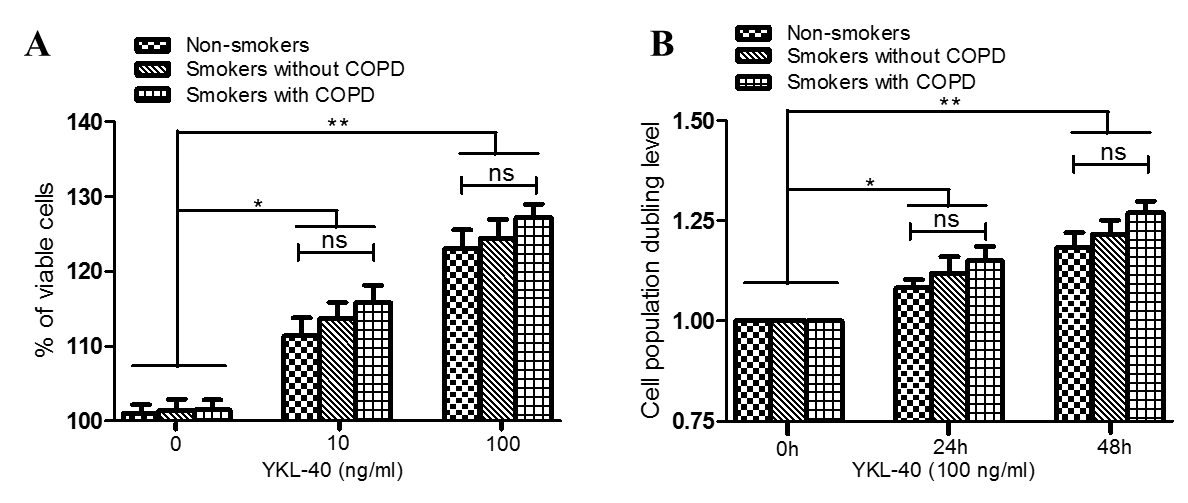


**Figure S1. The effect of YKL-40 treatment on the proliferation of human lung fibroblast cells from non-smokers, smokers without COPD and smokers with COPD.** The cells were treated with 0, 10 or 100 ng/ml YKL-40 for 48 h, then resuspended and counted (A); The cells were treated with 100 ng/ml YKL-40 for 0 h, 24 h and 48 h, then cell viability was measured with CCK-8 assay. Increased number of YKL-40-treated fibroblasts versus control fibroblasts was observed (B). (*p < 0.05, **p < 0.01 compared with basal). Results were expressed as mean ± SEM of three independent experiments.
